# Digital marketing of e-cigarettes in Southeast Asia: a neglected digital health and platform governance challenge for youth protection

**DOI:** 10.3389/fdgth.2026.1790973

**Published:** 2026-06-04

**Authors:** Myo Zin Oo, Soe Sandi Tint, Kong Sam An, Aung Tun, Kanittha Thaikla

**Affiliations:** 1 Research Institute for Health Sciences, Chiang Mai University, Chiang Mai, Thailand; 2 Global Health and Chronic Conditions Research Center, Chiang Mai University, Chiang Mai, Thailand; 3 Department of Family Medicine, Faculty of Medicine, Chiang Mai University, Chiang Mai, Thailand; 4 Department of Mental Health and Substance Abuse, Ministry of Health, Phnom Penh, Cambodia; 5 Tun Khit Foundation, Yangon, Myanmar

**Keywords:** digital health, digital marketing, e-cigarettes, platform governance, social media, Southeast Asia, youth

## Introduction

Rapid digitalization in Southeast Asia has fundamentally reshaped how young people communicate, socialize and consume commercial content, creating poorly regulated pathways for tobacco promotion. In parallel, exposure to electronic cigarettes (e-cigarettes) has increased among adolescents and young adults across the region (1). Although e-cigarettes continue to be framed within harm-reduction debates for adults who smoke, their digital marketing has emerged as an under-recognized threat to youth health (2). This risk is particularly pronounced in Southeast Asia, where youthful population structures, exceptionally high social media engagement and uneven regulatory and enforcement capacity converge to create a permissive environment for youth-oriented e-cigarette promotion (3). These dynamics position digital e-cigarette marketing as a critical digital health and platform governance challenge with direct implications for youth health behaviors.

To address this gap, this Opinion advances a conceptual framework that positions digital e-cigarette marketing as platform-mediated health communication within transnational digital ecosystems. It explains how platform features, social and perceptual processes, digital access pathways, and regulatory environments interact to shape youth susceptibility and use, providing a structured, mechanism-based lens for understanding youth vulnerability in Southeast Asia.

## Approach and scope of this opinion

This Opinion synthesizes empirical, review, and policy literature on digital e-cigarette marketing and youth exposure in Southeast Asia. It interprets the evidence through a digital health and platform governance lens to develop a conceptual framework and identify policy-relevant gaps.

## A conceptual framework: platform-mediated e-cigarette marketing and youth behavior

**Figure 1:**
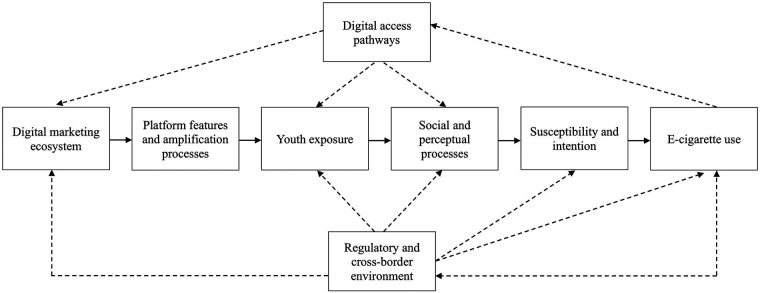
Conceptual framework of platform-mediated e-cigarette marketing and youth e-cigarette behavior in Southeast Asia. The framework illustrates how platform-mediated marketing shapes youth exposure, social and perceptual processes, and subsequent e-cigarette use, with digital access pathways and regulatory environments acting as cross-cutting influences. Arrows indicate conceptual relationships.

Digital marketing of e-cigarettes can be conceptualized as a platform-mediated health communication system (Figure 1) in which commercial actors, influencers, and users interact within algorithmically curated digital environments. Platform affordances, including content recommendation algorithms, engagement metrics, and network structures, shape the visibility, reach, and repetition of e-cigarette-related content.

We propose a structured pathway linking digital marketing to youth behavioral outcomes (Figure 1). First, the digital marketing ecosystem, operating through platform features and amplification processes, contributes to youth exposure to promotional and user-generated content. Second, exposure contributes to social and perceptual processes, including perceived norms, desirability, and reduced risk perception (2). Third, these processes shape susceptibility and intention to use. Finally, e-cigarette use may occur, often facilitated by digital access pathways, which also influence exposure and content interpretation. These processes are shaped by the regulatory and cross-border environment, influencing exposure, perception, and behavioral outcomes.

This framework conceptualizes youth e-cigarette use as an outcome of interactions between platform design, social influence, digital access pathways, and regulatory environments. It provides a structured lens to interpret evidence and identify intervention points, incorporating feedback mechanisms through which behavior reinforces access pathways and regulatory responses. Integrating social influence theory, health behavior models, and platform governance perspectives, the framework explains how digital marketing translates into behavioral outcomes, with arrows indicating conceptual rather than strictly causal relationships.

## Digital ecosystems of e-cigarette marketing and youth exposure

Digital marketing of e-cigarettes now operates through a platform-mediated ecosystem that extends beyond conventional advertising, encompassing influencer-style promotion, short-form content, user-generated content, and online sales channels (4). Evidence from Asian contexts, including India and Indonesia, demonstrates that these promotional practices persist even in jurisdictions with formal bans on e-cigarette sales or advertising, exposing a structural gap between regulatory intent and real-world enforcement (4, 5). Comparable platform-mediated marketing dynamics have been documented across Southeast Asia, including high adolescent exposure to online tobacco marketing in Indonesia (6), persistent social media-based promotion and sales of e-cigarettes in Thailand despite formal bans (7), and extensive lifestyle-oriented marketing on Malaysian e-cigarette retailer websites with frequent social media linkages (8).

Influencer-led promotion is particularly problematic, as it blurs the boundary between commercial messaging and peer communication, increasing the appeal and normalization of e-cigarette use within youth digital cultures (2). These dynamics reflect the initial stage of the proposed framework, where platform-mediated marketing shapes youth exposure to e-cigarette content.

## Digital marketing, social influence, and youth susceptibility to e-cigarette use

Systematic review evidence consistently links exposure to e-cigarette advertising and promotion, particularly via digital and social media platforms, with increased susceptibility, intention to use, and initiation among adolescents and young adults (2, 9). In Southeast Asia, youth-appealing e-cigarette marketing on social media platforms has been documented in the Philippines (10), while qualitative evidence from Singapore indicates that exposure to vaping-related social media content normalizes vaping and shapes more favorable perceptions despite comprehensive prohibitions (11). These associations appear stronger for digital media than for traditional advertising, reflecting the influence of social norms and repeated exposure within platform-mediated environments. Recent population-based evidence from Northern Thailand further supports this mechanism, showing that online information-seeking and perceived accessibility were strongly associated with e-cigarette use among adolescents and young adults, while perceived harm and household stigma were protective factors (1). Importantly, these patterns extend to young people who do not smoke, suggesting expansion of nicotine use beyond cessation contexts. Evidence from Southeast Asia remains limited but is growing, with increasing evidence on platform-mediated exposure and youth behavioral responses.

Within the proposed framework, these dynamics reflect the interaction between exposure and social and perceptual processes, through which repeated and socially mediated exposure shapes perceived norms, desirability, and risk perception, thereby contributing to susceptibility and intention to use e-cigarettes.

## Digitally mediated youth cultures and normalization of e-cigarette use

Qualitative research indicates that the use of e-cigarettes has become embedded within digitally mediated youth cultures, where commercial promotion, peer interaction, and identity formation increasingly overlap (12). In these environments, influencer-led and user-generated content normalize e-cigarette use through repeated, low-salience exposure rather than overt persuasion, making regulatory detection and digital enforcement more difficult. Empirical evidence from Southeast Asia documents the normalization of vaping within youth digital cultures, including through social media depictions of vaping in everyday social contexts and peer networks in settings such as Singapore and Thailand (7, 11). These dynamics are especially salient in Southeast Asia, where adolescents are among the most intensive users of major social media platforms, amplifying both the reach and impact of youth-oriented e-cigarette content. These processes embed e-cigarette use within everyday online practices, reducing its visibility as a health risk and increasing resistance to conventional prevention strategies. These processes reflect the social and perceptual stage of the framework, where repeated digital exposure shapes norms, desirability, and risk perception, increasing susceptibility and intention to use e-cigarettes.

## Transnational platforms and fragmented digital regulation

The risks posed by digital e-cigarette marketing are magnified by the transnational nature of online platforms. In Southeast Asia, this transnationality is evident in cross-border exposure to vaping-related content reported in Singapore, where participants encountered vape promotions and influencer posts originating from neighboring countries with less stringent regulatory environments, alongside extensive social media-based e-cigarette marketing documented in Thailand despite formal prohibitions (7, 11). Social network analyses show that e-cigarette influencers are embedded within dense, cross-border promotional networks, allowing marketing content to circulate seamlessly across jurisdictions and circumvent nationally defined regulatory controls (13). At the same time, regional policy reviews indicate that e-cigarette regulation across Southeast Asia remains highly fragmented, ranging from comprehensive bans to minimal oversight, with digital enforcement capacity consistently limited (3, 14).

Recent policy developments in Myanmar further illustrate evolving regional responses, with the introduction of a comprehensive national ban on e-cigarettes (15). However, such prohibitions expose limits of national regulation, as cross-border digital marketing and online sales persist. Consequently, tobacco control frameworks that remain nationally bounded are structurally ill-equipped to regulate a borderless, digitally mediated e-cigarette market. From a digital health governance perspective, this misalignment reflects a structural gap between nationally defined regulatory frameworks and platform-mediated environments that operate across jurisdictions. These dynamics reflect the framework's governance layer, where cross-border regulation shapes exposure, behavior, and policy effectiveness (Box 1).

BOX 1 **Why digital marketing of e-cigarettes poses unique risks for youth in Southeast Asia.**

Digital platforms function as powerful health communication infrastructures that algorithmically amplify e-cigarette promotion by prioritizing visually engaging and socially endorsed content, enabling influencer marketing and user-generated posts to reach large youth audiences across national borders. These platform features contribute to increased youth exposure and shape how e-cigarette-related content is encountered and interpreted. Evidence from social media monitoring and network analyses shows that such content persists despite formal regulatory restrictions, directly undermining country-level advertising and sales controls (4, 13).

High youth population shares and exceptionally intensive social media use in Southeast Asia further increase vulnerability to these exposures. Systematic reviews consistently demonstrate that digital e-cigarette marketing is associated with greater curiosity, more favorable attitudes, and increased intention to use e-cigarettes among adolescents and young adults, including those who have never smoked (2, 9). These patterns reflect the role of social and perceptual processes, including perceived norms and reduced risk perception, in shaping susceptibility to use.

These risks are compounded by fragmented regulatory frameworks and limited digital monitoring and enforcement capacity, which influence exposure, perception, and behavioral outcomes. Although the World Health Organization has repeatedly identified digital marketing, influencer promotion, and youth exposure as priority tobacco control concerns, implementation across Southeast Asia has lagged behind the rapid expansion of online and platform-mediated e-cigarette markets (16–18).

Source: Author’s synthesis and interpretation based on published empirical studies and World Health Organization guidance.

## Critical gaps and uncertainties in the evidence base

Despite growing evidence linking digital marketing to youth e-cigarette use, key gaps remain. Much of the literature is cross-sectional, limiting causal inference (2), and subject to self-reported measures and potential reverse causality. Longitudinal evidence on how platform-mediated exposure shapes social and perceptual processes, susceptibility, and use remains limited, particularly in Southeast Asia. Platform-level data are largely inaccessible, constraining understanding of exposure dynamics and the distinction between organic and commercially amplified content. Evidence is uneven across countries, and research on digital access pathways and enforcement strategies, including platform moderation and cross-border coordination, remains scarce. Addressing these gaps is essential for strengthening digital health governance responses.

## Discussion

The World Health Organization (WHO) has issued an explicit call to action on e-cigarettes, prioritizing the protection of children and adolescents and clearly identifying digital marketing, social media and influencer promotion as urgent regulatory concerns (16). WHO technical and regional analyses further document rapid market expansion, youth-targeted promotional strategies and persistent enforcement gaps, particularly in the Western Pacific and Southeast Asian regions (17, 18). These gaps are reflected in recent empirical evidence from Southeast Asia documenting persistent online e-cigarette promotion and youth exposure across multiple country contexts, including settings with formal prohibitions as well as those with limited digital regulatory enforcement (7, 8, 10, 11). From a digital health governance perspective, the continued visibility and normalization of digital e-cigarette marketing reflect a failure to translate global guidance into effective action, driven by the interaction between platform-mediated exposure, social and perceptual processes, and weak regulatory environments that collectively shape youth susceptibility and use.

Addressing digital e-cigarette marketing requires a recalibration of tobacco control strategies for digital environments. Regulatory frameworks must move beyond static, product-focused definitions of advertising to explicitly encompass influencer marketing, algorithmic amplification, and cross-border digital promotion. Priority actions include strengthening real-time digital surveillance and enforcement mechanisms, enhancing regional regulatory coordination, and embedding youth protection principles within broader digital governance and platform accountability frameworks. Without such measures, tobacco control gains risk erosion through the normalization of nicotine use in digital youth environments. Failure to address these platform dynamics risks leaving youth protection frameworks structurally misaligned with the digital realities through which contemporary health behaviors are formed. Interventions should address exposure, social drivers, access pathways, and regulatory coordination across jurisdictions. This highlights the need for a structured policy response to platform-mediated e-cigarette marketing, as outlined below.

Digital marketing of e-cigarettes is not a future concern but a current public health threat in Southeast Asia. Failure to confront this challenge risks entrenching nicotine use within digitally mediated youth cultures and undermining existing tobacco control policies. As digital platforms continue to shape how health-related norms, identities, and behaviors are formed among young people, regulatory inaction risks widening the gap between established tobacco control frameworks and contemporary digital realities. Without coordinated action, feedback between user behavior, access pathways, and regulation may reinforce exposure over time. A timely and coordinated response that addresses the realities of platform-driven promotion is therefore essential to protecting young people and sustaining tobacco control gains.

## Policy roadmap for youth protection

Addressing digital e-cigarette marketing requires coordination between tobacco control and digital governance. Building on the proposed framework (Figure 1), interventions should target key stages of the platform-mediated pathway. Regulatory definitions must expand to include influencer marketing, user-generated promotion, and platform-driven content amplification to reduce youth exposure. Regional coordination across Southeast Asia is essential to address cross-border marketing through shared standards, information exchange, and joint enforcement mechanisms. Interventions should also target the full marketing ecosystem, including brands, retailers, and content creators, by strengthening transparency requirements and restricting promotional practices that facilitate youth engagement. Digital platforms should be held accountable for limiting youth-targeted content through stronger age verification and content control measures. Real-time digital surveillance systems are needed to monitor evolving marketing strategies and support adaptive regulatory responses. Finally, youth-centered strategies, including digital media literacy and platform-based risk communication, should be integrated into tobacco control efforts to address social and perceptual drivers of susceptibility.
